# Frequency of psychological stress among women with new onset menstrual disorders amid corona pandemic lockdown

**DOI:** 10.12669/pjms.38.5.4606

**Published:** 2022

**Authors:** Amber Tufail, Rozina Mustafa, Sagheera Anjum Munaver, Beenish Nawaz

**Affiliations:** 1 Dr. Amber Tufail, FCPS. Professor, Department of Obstetrics & Gynaecology, Fazaia Ruth Pfau Medical College, Karachi, Pakistan; 2 Dr. Rozina Mustafa, FCPS. Professor & HOD, Department of Obstetrics & Gynaecology, Fazaia Ruth Pfau Medical College, Karachi, Pakistan; 3 Dr. Sagheera Anjum Munaver, FCPS. Associate Professor, Department of Obstetrics & Gynaecology, Fazaia Ruth Pfau Medical College, Karachi, Pakistan; 4 Dr. Beenish Nawaz, MSc, PhD. Assistant Professor, Department of Psychiatry, Fazaia Ruth Pfau Medical College, Karachi, Pakistan

**Keywords:** COVID-19, Dysmenorrhea, Menstrual Disorders, Psychological Stress, Premenstrual Syndrome

## Abstract

**Objectives:**

To determine the frequency of psychological stress and associated demographic factors among women with new onset menstrual disorders amid corona pandemic lockdown.

**Methods:**

This prospective cross-sectional study was conducted at the Pakistan Air Force Faisal Base Hospital, Karachi, Pakistan from 1^st^ April 2020 to 31^st^ July 2020. All women presented in the outpatient department or sought advice through telemedicine with new onset menstrual disorders during lockdown of COVID-19 were included. Information like age, parity, educational status, employment status, last menstrual period, previous cycle, new onset dysmenorrhea, and premenstrual syndrome were collected. Furthermore, level of stress was assessed by using 10 item Perceived Stress Sale questionnaire described by Sheldon Cohen in 1983.

**Results:**

Of total 916 women who consulted the department physically or through telemedicine, 59 (31.3%) had new onset disease. The frequency of different types of new onset menstrual problem were scanty menstrual flow 21 (35.6%), oligomenorrhea 6 (10.2%), menorrhagia 5 (8.5%), amenorrhea 4 (6.8%), and polymenorrhagia 2 (3.4%). New onset premenstrual syndrome and dysmenorrhea were present in 39 (66.1%) and 31 (52.5%) respectively. A significant association of premenstrual syndrome was observed with age (p-value 0.003), parity (p-value 0.045), educational status (p-value 0.007), and menstrual pattern (p-value 0.037). Furthermore, moderate stress was observed in 57 (96.6%) while severe in 2 (3.4%) patients.

**Conclusion:**

Among women presented with new onset menstrual disorders in gynecological outpatient, a considerable number reflected increased level of psychological stress during COVID-19 lock down.

## INTRODUCTION

Menstrual function is a significant factor of women’s psychological health. Stress can be a contributor condition to menstrual disorder^1^, which may include abnormal uterine bleeding and non-bleeding disorders like dysmenorrhea and premenstrual syndrome.

Menstrual health implies a healthy hypothalamus-pituitary ovarian axis and a normal uterus.^2^ Psychosocial or perceived stressors is defined as challenges that individuals view as taxing or exceeding their coping abilities lead to dysregulation.^3^

In Pakistan, first case of COVID-19 was reported in February 2020 and a month later lock down was imposed.^4^ During the pandemic, the disease itself, conspiracies, uncertainty, unemployment, isolations, and dubiousness led to many psychiatric epidemics concurrently globally.^5^ A recent meta-analysis has reported prevalence of stress, anxiety, and depression because of the pandemic in the general population significantly.^6^

Environmental stressors are of particular concern for women’s health, as they cannot only risk their mental health, but they can also adversely affect the reproductive outcome as well.^7^ A prospective cohort study has indicated that it is recent stress that has the greatest impact on menstrual cycle parameters, including ovulatory function.^3^

Review of literature showed many studies reporting a relationship between psychological stress and menstrual disorder. It is noticed that lockdown has increased this problem. As no such study was found in local perspective, we therefore planned this study in collaboration with psychologists, which will not only identify the new onset menstrual disorders and psychological stress during lock down, but women will also be provided counselling, prompt treatment of disorders and psychological support to cope up with the stress.

**Table I:** Comparison of premenstrual syndrome and dysmenorrhea with baseline characteristics among women with new onset menstrual disorder (n=59).

	Premenstrual Syndrome			Dysmenorrhea		
	Yes (n=39)	No (n=20)	p-value	Yes (n=31)	No (n=28)	p-value
**Age, years**						
≤25	16 (76.2)	5 (23.8)	0.003	13 (61.9)	8 (38.1)	0.013
26-40	21 (75)	7 (25)		17 (60.7)	11 (39.3)	
>40	2 (20)	8 (80)		1 (10)	9 (90)	
**BMI**						
Underweight	10 (83.3)	2 (16.7)	0.248	6 (50)	6 (50)	0.814
Normal	14 (73.7)	5 (26.3)		11 (57.9)	8 (42.1)	
Overweight	10 (55.6)	8 (44.4)		10 (55.6)	8 (44.4)	
Obese	5 (50)	5 (50)		4 (40)	6 (60)	
**Marital Status**						
Unmarried	18 (75)	6 (25)	0.232	16 (66.7)	8 (33.3)	0.072
Married	21 (60)	14 (40)		15 (42.9)	20 (57.1)	
**Parity (n=35)**						
Nulliparous	3 (100)	0 (0)	0.045	2 (66.7)	1 (33.3)	0.560
Primiparous	10 (83.3)	2 (16.7)		6 (50)	6 (50)	
Multiparous	6 (40)	9 (60)		6 (40)	9 (60)	
Grand Multiparous	2 (40)	3 (60)		1 (20)	4 (80)	
**Education**						
Primary or less	1 (16.7)	5 (83.3)	0.007	1 (16.7)	5 (83.3)	0.166
Intermediate or less	17 (85)	3 (15)		12 (60)	8 (40)	
Graduate or more	21 (63.6)	12 (26.4)		18 (54.5)	15 (45.5)	
**Occupation**						
Unemployed	21 (65.6)	11 (34.4)	0.933	15 (46.9)	17 (53.1)	0.343
Employed	18 (66.7)	9 (33.3)		16 (59.3)	11 (40.7)	

Asian criteria-based BMI was used: <18.5 for underweight, 18.5-22.9 for normal-weight, 23.0-27.5 for overweight, and >27.5 for obese women. Fisher-exact test, chi-square test, p-value <0.05 considered significant.

## METHODS

This cross-sectional study included patients who contacted outpatient or tele clinic of Pakistan Air Force Hospital Faisal Base, Karachi due to new onset menstrual disorder and consented to participate. Ethical approval was granted from the institute.(Ref.002 dated October 3, 2020) Data was collected from the 1^st^ April to 31^st^ July 2020. Women with menstrual disorders in the preceding 6-month, age less than 20 years or more than 45 years, breast feeding, on hormonal contraception, and with known medical condition were excluded.

Epi Info sample size calculator is used for the estimation of sample size taking confidence interval 95%, margin of error 8%, frequency of menstrual disorder during COVID-19 in previous study 10.5%.^8^ The estimated sample size came out to be 56 patients with new onset menstrual disorder at least. The term dysmenorrhea was defined as painful menstruation. A cycle duration of more than 35 days was labeled as Oligomenorrhea whereas lesser than 21 days was labeled as Polymenorrhea. Excessive flow (>5 pads/day) and/or length (>5 days) were used to describe menorrhagia. Hypomenorrhea (scanty periods) was described as <3 days period or the use of <two pads per day. The absence of menstruation for three cycles was considered amenorrhea. Premenstrual syndrome was characterized as any symptom that occurs 5-10 days before menstruation and disappears after menstruation, such as painful/tender breasts, bloating/swelling of the abdomen, mood changes, depression, or others.

The questionnaire was developed by the principal investigator and was divided into three parts. The first encompassed the demographic data including age, parity, educational and employment status. The second part comprised of anthropometric measurements including height, weight, and BMI. The third part, menstrual history, included last menstrual period, previous cycle, dysmenorrhea, and premenstrual syndrome. Pilot testing of the questionnaire was done to check its sequencing, phrasing, and understanding. Level of stress was determined by psychiatrist by filling the Perceived Stress Sale questionnaire.^8^ There were 10 questions with five-point scale (0=never, 1=Almost never, 2=sometimes, 3=fairly often, 4+very often). Six items of this scale, i.e., 1, 2, 3, 6, 9 and 10 were scored in an ascending order and the rest of seven items, i.e., 4, 5, 6, 7 & 8 were scored in descending order. The total score on PSS was taken as sum of score for all the 10 items.

Statistical analysis was performed using SPSS version 24. Descriptive statistics were explored using frequencies and percentages. While for the purpose of inferential statistics, Chi-square test/Fisher Exact test were applied. The p-value of <0.05 is considered as significant.

## RESULTS

Out of 916 gynecological consultations 59 (6.44%) were due to new onset menstrual disease. Among them 28(47.5%) belonged to age range of 26-40 years, 35(59.3%) were married and 15(42.9%) were multiparas.

A significant association of premenstrual syndrome was observed with age (p-value 0.003), parity (p-value 0.045), educational status (p-value 0.007), and menstrual pattern (p-value 0.037). However, dysmenorrhea was found significantly associated with age of the patients (p-value 0.013) and history of COVID-19 positivity only (p-value 0.010). (Table-II& III)

**Table II:** Comparison of premenstrual syndrome and dysmenorrhea with clinical characteristics among women with new onset menstrual disorder (n=59).

	Premenstrual Syndrome			Dysmenorrhea		
	Yes (n=39)	No (n=20)	p-value	Yes (n=31)	No (n=28)	p-value
**Weight gain**						
None	16 (64)	9 (36)	0.332	13 (52)	12 (48)	0.641
Up to 5 kg	19 (63.3)	11 (36.7)		15 (50)	15 (50)	
More than 5 kg	4 (100)	0 (0)		3 (75)	1 (25)	
**Menstrual Pattern**						
Regular	15 (71.4)	6 (28.6)	0.037	13 (61.9)	8 (38.1)	0.062
Scanty	13 (61.9)	8 (38.1)		9 (42.9)	12 (57.1)	
Amenorrhea	0 (0)	4 (100)		0 (0)	4 (100)	
Oligomenorrhea	4 (66.7)	2 (33.3)		3 (50)	3 (50)	
Polymenorrhea	2 (100)	0 (0)		1 (50)	1 (50)	
Menorrhagia	5 (100)	0 (0)		5 (100)	0 (0)	
No	10 (62.5)	6 (37.5)		4 (25)	12 (75)	

Fisher-exact test, chi-square test, p-value <0.05 considered significant.

**Table III:** Findings of the perceived stress scale score in women with new onset menstrual disorders^8^ (n=59).

	Never	Almost Never	Sometimes	Fairly Often	Very Often

	n (%)	n (%)	n (%)	n (%)	n (%)
Been upset because of something that happened unexpectedly	0 (0)	3 (5.1)	12 (20.3)	25 (42.4)	19 (32.2)
Unable to control the important things in life	1 (1.7)	6 (10.2)	12 (20.3)	25 (42.4)	15 (25.4)
Felt nervous and “stressed”	1 (1.7)	2 (3.4)	10 (16.9)	23 (39.0)	23 (39.0)
Felt confident about ability to handle personal problems	2 (3.4)	2 (3.4)	15 (25.4)	30 (50.8)	10 (16.9)
Felt that things were going away	2 (3.4)	6 (10.2)	16 (27.1)	24 (40.7)	11 (18.6)
Could not cope with all the things that had to do	1 (1.7)	7 (11.9)	14 (23.7)	22 (37.3)	15 (25.4)
Been able to control irritations in life	3 (5.1)	5 (8.5)	24 (40.7)	19 (32.2)	8 (13.6)
Felt on top of things	1 (1.7)	9 (15.3)	21 (35.6)	23 (39.0)	5 (8.5)
Angered because of things that were outside of control	1 (1.7)	7 (11.9)	21 (35.6)	21 (35.6)	9 (15.3)
Felt difficulties were piling up so high that could not overcome	1 (1.7)	4 (6.8)	24 (40.7)	23 (39.0)	7 (11.9)

**Fig.1:**
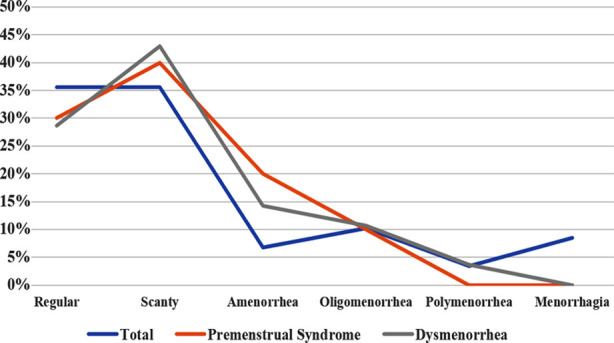
Line graph showing menstrual pattern in all women with new onset menstrual disorder and also with respect to premenstrual syndrome and dysmenorrhea (n=59).

The frequency of new onset menstrual problems noted were scanty periods 21 (35.6%), oligomenorrhea 6 (10.2%), menorrhagia 5 (8.5%), amenorrhea 4 (6.8%), and Polymenorrhea two (3.4%) (Fig.1).

Most of the women with new onset menstrual disorder reported “very often” for felt nervous and stressed 23 (39%) and upset 19(32.2%) in the past last month. Moderate stress was observed in 57 (96.6%) while severe in 2 (3.4%) patients (Fig.1). A non-significant association of stress was observed with menstrual pattern (p-value 0.587), premenstrual syndrome (p-value 0.625) and dysmenorrhea (p-value 0.942).

## DISCUSSION

The current study was aimed to determine frequency of new onset menstrual disorder and level of psychological stress among these women during lockdown phase of COVID-19 pandemic. Overall, we found high prevalence of stress among participants, majority were moderately and upto three percent were severely stressed. Similarly, a cross-sectional online survey involving front-line health care providers in India has reported almost four percent prevalence of high stress among them.^9^ The current study has explored the fact reproductive aged group women who seek gynecological consultation had stress equivalent to a frontline health care worker. Pakistan has been identified as a country with highest scores of stresses next to Canada and the United Kingdom during Covid pandemic.^10^

Almost six percent of gynecological consultation were due to new onset menstrual disease burden. Earlier, outside the pandemic a much higher proportion of menstrual irregularity in outpatients was reported.^11^ Our reported frequency is much less as compared to the above mentioned study because have excluded a large number of women in view of our exclusion criteria. In the current study, insignificant p-values were observed between psychological stress and menstrual irregularities, premenstrual syndrome, and dysmenorrhea. Similarly, another study involving undergraduate medical students failed to determine association between psychological stress and menstrual abnormalities.^12^ Various studies have explored an association between psychological stress and menstrual abnormalities including premenstrual syndrome and dysmenorrhea.^13^-^15^ The mixed results highlight the disparity of evidence that exists in the literature regarding the association between stress and menstrual abnormalities.

The most commonly reported menstrual irregularity in this study was hypomenorrhea. In contrast, different associations were explored in other studies. A higher level of perceived stress was found to be associated with a higher prevalence of shorter cycle length and heavy bleeding.^2^ Another study conducted on hostellers of a medical college had found an association between high stress levels and irregular menstrual cycle.^16^ One reason for this difference in findings was the reduced sample size in our study as compared to the other. Moreover, in our study we have excluded all the women who have menstrual disorder in the preceding six months and included women belonging to a broader age range 20-45 years.

In the current study, premenstrual syndrome was observed in 66.1% of women. A significant association of premenstrual syndrome was found with age, parity, educational level, and menstrual pattern. Earlier, a study involving women of different sociodemographic strata has determined association between PMS and high education. Although the distribution of symptomatology differs in different age and parous group but failed to identify at risk age and parous group.^17^ An insignificant p-value was calculated between weight and premenstrual syndrome in the current study. Similar finding was determined by another study.^18^ In contrast, the risk of premenstrual syndrome was reported to be 8.2% higher in obese women as compared to underweight women in one study.^19^ The reason for this difference is in the age of participants. The aforementioned study was on adult women whereas a broader age group from 20-45 years in the current study.

Prevalence of dysmenorrhea reported in this study was 52.5%. The range of 45%-97% was reported from community-based studies in the WHO review. In this study, the reported prevalence stands at the lower limit. The reason for this difference is that we rely on data taken from tele clinic during the pandemic and a broader age range. A significant p-value was calculated between dysmenorrhea and age of the patient in this study. Similarly, studies done in the past have documented an inverse relationship and risk of dysmenorrhea. Menstrual characteristics of females are known to be risk factors for dysmenorrhea. In this study an insignificant association of dysmenorrhea with menstrual pattern was determined. However, a study done in the past has determined that heavy menstrual bleed is risk factor for dysmenorrhea and its severity.^20^ In the current study menorrhagia was present in five cases but none has associated dysmenorrhea. A possible explanation is the limited sample size in this study and unreliable inference.

Female gender is identified at risk for psychological stress which tend to be exacerbated in stress like a pandemic.^21^ This study is unique in the sense that it involves determining psychological stress in special subset of women requiring consultation due to gynecological issues amid the COVID pandemic. There is a need to address menstrual health and develop stress reducing strategies in such situations in the future.

### Limitations of the study:

The main limitation is small number of participants; stress was assessed only subjectively and participants were not followed. Physiological biomarkers of stress like serum or salivary cortisol level were not measured.

## CONCLUSION

The findings revealed that women who seek gynecological consultation either physically or virtually during lock down of COVID-19 pandemic were subjected to increased level of psychological stress. The COVID-19 pandemic affects menstrual health, and targeted interventions are needed to improve it. Future studies that explore psychological stress and menstrual disorders should include both psychological stress indices and measure biological markers of stress and simultaneously to determine causal biologic mechanism.

### Authors’ Contribution:

**AT:** Conceived, designed, data collection and manuscript writing.

**RM & SAM:** Did data collection its analysis and critical review of manuscript.

**BN:** Evaluate the psychological stress.
